# Which factor contribute most to empower farmers through e-Agriculture in Bangladesh?

**DOI:** 10.1186/s40064-016-3443-3

**Published:** 2016-10-07

**Authors:** Sheikh Mohammed Mamur Rashid, Md Rezwan Islam, Md. Quamruzzaman

**Affiliations:** 1 Department of Agricultural Extension and Information System, Sher-e-Bangla Agricultural University, Sher-e-Bangla Nagar, Dhaka, 1207 Bangladesh; 2 Department of Finance, University of Dhaka, Dhaka, 1000 Bangladesh; 3 Department of Agronomy, Sher-e-Bangla Agricultural University, Sher-e-Bangla Nagar, Dhaka, 1207 Bangladesh

**Keywords:** e-Agriculture, Farmer’s empowerment, Usages of agricultural technology, Modern agriculture, Agricultural extension, Digital Bangladesh

## Abstract

The purpose of this research was designed to investigate the impact of e-Agriculture on farmers of Bangladesh. Empowerment is stratified as economic, family and social, political, knowledge and psychological empowerment. Data were collected in Bhatbour Block of Dhighi union under Sadar Upazila of Minikganj District. Data were collected in two phases from the same group of respondents (in August, 2013 and September, 2015). Two sample t test and step-wise multiple regression method were used for analysis. The results showed that e-Agriculture had significant impact on the empowerment of farmers of Bangladesh. Additionally, the study concluded that the most significant factor behind the empowerment of farmer was the use of e-Agriculture which could explain almost 84 % of the total variation of the empowerment. Based on the findings, it is recommended that government should implement e-Agriculture based projects on a massive scale for the empowerment of the farmers.

## Background

e-Agriculture as an emerging field in the intersection of agricultural informatics, agricultural development, and entrepreneurship, referring to agricultural services, technology dissemination, and information delivered or enhanced through the Internet and related technologies (FAO 2005). The application of e-Agriculture is still in its elementary stage, evolving around the immense multiplier impact capability that can significantly change the farmer’s economic and social condition i.e. empowerment. This ensures the effective and efficient use of information and communication technologies for analyzing, designing and implementing existing and innovative applications to help the agricultural sector. In 2008, Bangladesh Institute of ICT in Development (BIID), in collaboration with Katalyst (Partner of Swiss Contact and a local agro-based NGO) and Grameenphone launched the e-krishok initiative (New Agriculturist 2015). The purpose of these project was to lessen the information inadequacy in the agriculture sector and thus enabling the farmers with up-to-date knowledge and advisory services which they often required. After that, Bangladesh government came up with the idea of “Digital Bangladesh” with a vision to leverage the power of ICT in each and every public sector and service (A2i 2014). Keeping that in mind, Government Launched several projects to digitalize the agricultural services as well in empowering the farmers. Empowerment is a process of change by which individuals or groups gain the power and ability to take control of their lives (World Bank 2011). Therefore, this idea of farmer’s empowerment by the means of e-Agriculture has been studied to find out whether the initial wave of e-Agriculture attempts made some productive impacts or not.

## Literature review

The concept of e-Agriculture is still in the nascent stage in Bangladesh context, so does it in the academic arena. In 2003, under the “Support to ICT” taskforce program the ministry of agriculture of Bangladesh did set up an agricultural information system. (MoA 2003). In 2005, a group of researchers of D.Net (Development Research Network, Bangladesh) proposed the idea of “Pallitathya Help Center” and conducted a project on it. The idea centered on the use of relatively less fashionable ICT, the mobile phone, as an effective ‘last mile solution’ to improve access to livelihood information for the rural people. They found it most challenging to understand the problems (related to health, agricultural, weather information) of rural people and to provide the appropriate information (Raihan et al. 2005). Since this idea is brand new, this researcher has not come across any local literature that has made any qualitative attempt to measure the impact of e-Agriculture in the empowerment process. Hence, the quest for previous quantitative approached literature has been shifted to South Asian literatures because these countries share the similar socio-economic context. Ironically, this attempt has turned into a futile one also, as there are numerous literatures that have examined the women empowerment, economic empowerment through micro-finance but nothing in the field of farmer empowerment or impact of e-Agriculture. Out of all the literatures that have been reviewed, the researcher has found the literature of Sendilkumar (2012) from Kerala Agricultural University which has close match to the purpose of this literature. Sendikumar (2012) made an attempt to study the empowerment dynamics of kerala farmers who joined the grouped approached farming of Paddy introduced by the kerala local government. In this regard, he developed an Empowerment Dynamics Index (EDI) and computed the index for the before and after joining situation of these farmers. The result showed that this program had statistically significant role to set up sustainable development of the farmers in this state. So, this literature has been attempted in this Greenfield segment and perhaps the first of its kind in Bangladeshi context. Not to mention, the researcher has thoroughly gone through the other empowerment literatures from which statistical the model used in the context, has been applied.

## Methods

### Study location

The researcher applied purposive sampling technique to determine the location form where the data were collected. The study was conducted at the Bhatbour block of Dighi union under Manikganj Sadar Upazila, Manikganj (One of the major districts of Bangladesh) where the government of Bangladesh has been implementing a numbers of e-Agriculture related development projects with the help of foreign aids through Department of Agricultural Extension (DAE).

### Targeted population

For the purpose of this study, the farmers (within this block) those who used e-Agriculture were considered as the study group and the farmers those who did not use such (within this block) were considered as the control group. According to the DAE database, in this area, approximately 1148 farmers used e-Agricultural facilities.

### Sampling procedure

To determine the sample size out of these 1148 study group farmers, the researcher used Yamane’s (1967) formula:

$${\text{n}} = \frac{{{\text{z}}^{2} P \,(1 - P)N}}{{{\text{z}}^{2} P \,(1 - P) + N ({\text{e}})^{2} }}$$

where, n = Sample size; N = Population size = 1148; e = The level of precision = 8 %; z = the value of the standard normal variable given the chosen confidence level (e.g., z = 1.96 with a confidence level of 95 %) and P = The proportion or degree of variability = 50 %. According to the formula, the desired sample size (n) was = 133.

Thereafter, the desired respondents’ size of the control group was determined as 45. As the study group’s sample size was one third of its population, this same ratio was applied here to determine the control group sample size. After determining both of the sample sizes for each of the group, a semi-structured questionnaire was developed and printed for conducting one to one interview. To reduce information distortion, one farmer from each of the farming family was included in the survey. Furthermore, to ensure similar socio-economic conditions for both the control and test groups, a two-way stratified random sampling technique was used, in which education and farm size were considered as two individual strata. Education was further categorized into three groups: group 1 (denoted as E_1_), whether respondents were illiterate or could sign only; group 2 (denoted as E_2_), whether respondents had primary education or not, and group 3 (denoted as E_3_), whether respondents had secondary or higher. After that, Farm size was also categorized into three groups: group 1(denoted as F_1_), small farm group (farm size up to 0.5 hectors); group 2 (denoted as F_2_), medium-farm group (farm size 0.51–1.0 hector), and group 3 (denoted as F_3_), large farm group (farm size above 1.0 hector). The two-way stratified random table is given as Table 1.

**Table 1:** Two-way stratified random sampling of respondents based on their level of education and farm size

Category	% of respondents	No of respondents from the study group	No of respondents from control group (one-third of the study group)
E_1_ × F_1_	10.53	14	5
E_1_ × F_2_	5.26	7	3
E_1_ × F_3_	4.51	6	2
E_2_ × F_1_	21.05	28	9
E_2_ × F_2_	9.02	12	4
E_2_ × F_3_	12.03	16	5
E_3_ × F_1_	22.56	30	10
E_3_ × F_2_	9.02	12	4
E_3_ × F_3_	8.27	11	3
Total	100	133	45

With the help of the two-way stratified random sampling procedure, homogeneous/similar categories of control and testing group respondents were selected, and then the proportionate random sampling technique was used to select either study or control group respondents from each village. Data were collected in two phases from the same group of respondents (in August, 2013 and September, 2015). A reserve list was maintained to fill in the gaps if any respondent in the original list was found missing as the same respondent in two interviews (in August, 2013 and September, 2015). To ensure the same respondents for the two phase interviews, 5 % extra respondents were interviewed in the first phase and in the year of 2013 to fill in the gaps in case of any interviewed respondent unavailability in the second phase and in the year of 2015 interview period. The definitions of the variables measured are shown in Table 2.

**Table 2:** Variable measurement techniques

Category	Scoring system				
Age	1 for each complete year of age of the respondent				
Education	1 for each year of school education				
Effective farm size	1 for each decimal area of land				
Annual household income	1 for each “thousand BDT” income in a year				
Farming experience	1 for each year experience				
Participation in training	1 for each day training				
Agricultural knowledge	1 for each question’s correct answer and “0” for wrong answer				
Usages of e-Agriculture	Extent of uses				
	4 for frequently	3 for regularly	2 for occasionally	1 for rarely	O for not at all
Attitude towards e-Agriculture	Extent of opinion				
	(+2) for strongly agree	(+1) for agree	(0) for undecided	(−1) for disagree	(−2) for strongly disagree
Organizational participation	Nature of participation (years)				
	4 for President/	3 for secretary	2 for executive member	1 for ordinary member	0 for no participation
Cosmopoliteness	Places of visiting (years)				
	4 for frequently	3 for regularly	2 for occasionally	1 for rarely	O for not at all
Availability of e-Agriculture	Availability score				
	4 for frequently	3 for regularly	2 for occasionally	1 for rarely	O for not at all

### Minimizing spill-over effects

The study used a quasi-experimental survey design to resolve the problems of endogeneity both at location level and participant level. To overcome the transmission/contamination of information or knowledge from e-Agriculture users to non-users, i.e. diffusion of treatment, and to avoid downward bias, all control respondents were selected from those villages where e-Agriculture services had not introduced at all. These selected villages were exclusively surveyed by the study programs, where no organization(s) implemented a similar program within the villages, or even outside the villages within a considerable surrounding area. Moreover, a large distance (about 3–5 km) was maintained between the study and control group villages within the block (Hulme 2000). The study and control group respondents were also selected to represent both the Muslim and Hindu communities; between the nearest two groups, if one group contained a Muslim community, the other contained a Hindu community (Duvendack et al. 2011).

### Data collection

Data were collected personally by the researchers themselves through personal interview schedule from the sampled farm families of the selected areas. Before starting the collection of data, the researchers met the respective Upazila Agriculture Officer (UAO), Agriculture Extension Officer (AEO), Upazila Food Program Officer (UFPO), Assistant Health Inspector (AHI) and the concerned SAAOs. The researchers also discussed the objectives of the present study with the respondents and above mentioned officers and requested them to provide actual information. A rapport was established with the rural people so that they feel easy to answer the questions. The researchers took all possible care to establish rapport with the respondents so that they would not feel any indecision while starting the interview. A very good cooperation was obtained from the field extension workers and the local leaders. No serious difficulty was faced by the researchers during the collection of data. The interviews were made individually in the houses of respondents. Questions were asked in different ways so that the respondents could easily understand the questions. Whenever a respondent faced difficulties in understanding any questions, care was taken to explain the same clearly with a view to enabling him/her to answer it properly.

Before going to the respondents’ home for interviewing they were informed verbally to ensure their availability at home as per schedule date and time. In the case of failure to collect information from the respondents due to their other business, a revisit was made with prior to appointments. If any respondent failed to understand any question, the researchers took great care to explain the issue. If the respondents could not clear about what was wanted to know then supplementary questions were asked for further clarification. The researcher received full cooperation from the respondents during the time of interview. Data of both studied were collected August, 2013 and September, 2015, respectively.

### Empowerment Condition Index

To assess the impact of e-agriculture on farmer’s empowerment, the researcher designed a new model taking five factors into consideration: economic, family and social, political, knowledge and psychological empowerment and combines them to gauge the overall effect of e-Agriculture. Each parameter was developed by the outputs of focused group discussion with the officials, experts, academicians and experienced farmers. The impact/change of all the related variables were counted using a numeric value and if required, an equivalence factor was adjusted with the counted score, considering the number of members in study group and control group.

As the empowerment was measured by determining the five empowerment indicators, presented as the Empowerment Condition Index (ECI), the detail breakdown of each indicator is defined as such: (a) changes in economic empowerment, consists of income due to yield obtaining, saving money, investments, availing agriculture loans and purchase of farming inputs, (b) changes in family and social empowerment, considered by measuring changes in a respondent’s developing institutional contact, linkage with development departments, team spirit, leadership quality, group consensus to solve problem, (c) changes in political empowerment, where political empowerment was considered through changes in level of social well-being activities, membership in the social organization, freedom of expression and conflict management. (d) changes in knowledge empowerment, considered by measuring changes in a respondent’s use of machineries and equipment, knowledge on value addition, adoption of IPM, INM, IWM practices and (e) changes in psychological empowerment, considered by measuring changes in a respondent’s motivation in farming, self-esteem, risk taking ability, confidence and decision making ability. The respondents’ responses were counted by providing a score based on a response scale. Each respondent’s total change (unit free score) was considered as the ‘Empowerment Condition Index’.

### Statistical analysis

Data collected from the respondents were analyzed and interpreted in accordance with the objectives of the study. The analysis of data was performed using statistical treatment with SPSS (Statistical Package for Social Sciences) computer program, version 20. Statistical measures as a number, range, mean, standard deviation were used in describing the variables whenever applicable. In order to estimate the contribution of the selected characteristics of farmers in empowering them through e-Agriculture, step-wise multiple regression analysis (B) analysis was used. Throughout the study, 5 % (0.05) level of significance was used as the basis for rejecting any null hypothesis. If the computed value of (B) was equal to or greater than the designated level of significance (*p*), the null hypothesis was rejected and it was concluded that there was a significant contribution between the concerned variable. Whenever the computed value of (B) was found to be smaller at the designated level of significance (*p*), the null hypothesis could not be rejected. Hence, it was concluded that there was no contribution of the concerned variables. Changes in basic rights and changes in quality of life were considered as the dependent variables in order to develop step-wise multiple regression models for identifying related factors and their level of contribution to improving respondents’ empowerment conditions. The model used for this analysis can be explained as follows:

$${\text{Y}} = {\text{a}} + {\text{b}}_{1} {\text{x}}_{1} + {\text{b}}_{2} {\text{x}}_{2} + {\text{b}}_{3} {\text{x}}_{3} + {\text{b}}_{4} {\text{x}}_{4} + {\text{b}}_{5} {\text{x}}_{5} + {\text{b}}_{6} {\text{x}}_{6} + {\text{b}}_{7} {\text{x}}_{7} + {\text{b}}_{8} {\text{x}}_{8} + {\text{b}}_{9} {\text{x}}_{9} + {\text{b}}_{10} {\text{x}}_{10} + {\text{b}}_{11} {\text{x}}_{11} + {\text{b}}_{12} {\text{x}}_{12} + {\text{e}};$$

where Y = empowerment of farmers, of the independent variables, x_1_ is the respondent’s age, x_2_ is education, x_3_ is farm size, x_4_ is annual household income, x_5_ is farming experience, x_6_ is participation in training, x_7_ is agricultural knowledge, x_8_ usages of e-Agriculture, x_9_ is the attitude towards e-Agriculture, x_10_ is organizational participation, x_11_ is cosmopoliteness, x_12_ is the availability of e-Agriculture. b_1_, b_2_, b_3_, b_4_, b_5_, b_6_, b_7_, b_8_, b_9_, b_10_, b_11_ and b_12_ are regression coefficients of the corresponding independent variables, and e is random error, which is normally and independently distributed with zero mean and constant variance.

## Results and discussion

### A comparison between the study group and control group

A comparison between the Study Group (SG) and Control Group (CG) was done to find out whether or not e-Agriculture had substantial contribution towards farmers’ empowerment. The distributions of changed empowerment with respect to study group and control group respondents are shown in Table 3 along with t test (1 % level of significance) value.

**Table 3:** Distribution of study group and control group respondents’ level of empowerment based on their changed value

Sub-parameter of empowerment (scoring method)	Empowerment indicator	Study group (changed mean value differences)	Control group (changed mean value differences)	t test value
Economic empowerment	Increased income due to yield obtaining	0.955	0.674	3.728**
	Saving money	1.271	0.891	6.080**
	Investments	1.271	0.717	5.295**
	Availing agriculture loans	1.459	0.891	4.347**
	Purchase of farming inputs	1.248	1.217	3.162**
Sub total		6.203	4.391	
Family and social empowerment	Developing institutional contact	0.977	0.717	2.789**
	Linkage with developing departments	0.895	0.652	1.848
	Team spirit	1.105	0.761	6.514**
	Leadership quality	1.218	0.869	3.919**
	Group consensus to solve problem	1.293	0.783	5.449**
Sub total		5.488	3.781	
Political empowerment	Participation in social well-being activities	0.744	0.608	2.874**
	Membership in the social organization	0.406	0.456	0.724
	Freedom of expression	1.188	0.761	4.023**
	Conflict management	1.218	0.826	2.874**
Sub total		3.556	2.652	
Knowledge empowerment	Use of machineries and equipments	0.939	0.522	4.933**
	Knowledge on value addition	1.195	0.783	4.739**
	Adoption of IPM practices	1.316	0.848	4.392**
	Adoption of INM practices	1.188	1.217	2.031
	Adoption of IWM practices	1.226	0.957	3.511**
Sub total		5.864	4.326	
Psychological empowerment	Motivation in farming	0.939	0.587	3.697**
	Self esteem	1.015	0.739	4.057**
	Risk taking ability	1.181	0.935	3.500**
	Confidence	1.218	0.869	3.748**
	Decision making ability	1.105	1.131	0.553
Sub total		5.458	4.261	
Total		26.569	19.411	

** Significance at the level of 1 % (t-value)

### Calculation of empowerment impact

$$\begin{aligned} & {\text{Empowerment}}\,{\text{impact}}\,{\text{difference}} \\ & \quad = {\text{Mean}}\,{\text{score}}\,{\text{of}}\,{\text{study}}\,{\text{group}}\,{\text{empowerment}} - {\text{Mean}}\,{\text{score}}\,{\text{of}}\,{\text{study}}\,{\text{group}}\,{\text{empowerment}} \\ & \quad = 26.569 - 19.411 \\ & \quad = 7.158 \\ \end{aligned}$$

According to the table, there was a significant difference between study group and control group respondents’ level of empowerment based on t test statistics (1 % level of significance) value. So, there was a positive impact of e-Agriculture on farmers’ empowerment.

Since the comparison revealed e-Agriculture’s substantial impact on farmers’ empowerment, the researchers investigated further to identify the factors within the five parameters which changed significantly due to the involvement of e-Agriculture towards the empowerment of the study group.

### Economic empowerment

The economic empowerment of the farmer members was studied based on the selected parameters like income, savings habit, investments, financial management skill, extent of dependency on money lenders, purchasing of input of farming etc. and given in Table 4.

**Table 4:** Economic empowerment of farmers through e-Agriculture

Sl. no.	Economic empowerment components	Mean score		t test value
		Before e-Agriculture use	After e-Agriculture use	
1.	Increased income due to yield obtaining	2.47	3.43	15.949**
2.	Saving money	2.14	3.41	7.030**
3.	Investments	1.89	3.16	4.460**
4.	Availing agriculture loans	1.95	3.41	3.333**
5.	Purchase of inputs of farming	1.35	2.60	0.864
Total mean score		9.80	16.01	
Overall mean score		1.96	3.202	

** Significance at the level of 1 % (t-value)

It was observed that income of the respondents had been increased, which might be due to the increase in the yield obtained. The purchase of inputs for farming, respondents have gained increased mean score (2.60) especially after joining to the group, because of the reason that the required farm inputs information were provided by e-Agriculture at a subsidized cost. With respect to availing of agricultural loans, farmers had been empowered considerably (mean score 1.95–3.41), due to reason that, e-Agriculture was providing the information low or interest free loans to the farmers. The savings of the members had also increased (2.14) in spite of poor return from farming. From the data (Table 4), the t test also supported the obtained mean score and shown a significant difference.

Since the economic empowerment was positively significant to empower the farmers, the researchers investigated further to identify the family and social empowerment factor.

### Family and social empowerment

The family and social empowerment was studied in terms of freeness to work with group members, involvement in the decision making process, team spirit, leadership quality and group consensus to solve problem. The result depicted in Table 5.

**Table 5:** Family and social empowerment of farmers through e-Agriculture

Sl. no.	Family and social empowerment components	Mean score		t test value
		Before e-Agriculture use	After e-Agriculture use	
1.	Developing institutional contact	2.18	3.15	13.005**
2.	Linkage with developing departments	2.51	3.39	13.658**
3.	Team spirit	1.88	2.99	6.398**
4.	Leadership quality	1.96	3.18	6.291**
5.	Group consensus to solve problem	1.59	2.90	2.150
Total mean score		10.12	15.61	
Overall mean score		2.024	3.122	

** Significance at the level of 1 % (t-value)

From the Table 5, it was evident that the contact with institutions and linkage with development departments by the respondents had shown remarkable improvement. After usages of e-Agriculture, the mean scores for the above said subcomponents were increased from 2.18 to 3.15 and 2.51 to 3.39 respectively. Regarding group consensus to solve problem, there was an increase in mean score (1.59–2.90) noticed. It can be seen that team spirit and leadership quality of the respondents were improved. Data showed that, the t test also supported the obtained mean score and shown a significant difference.

Since the family and social empowerment factor was positively significant to empower the farmers, the researchers investigated further to identify the political empowerment factor.

### Political empowerment

The political empowerment studied with variables like participation in social well-being activities, membership in social organization and conflict management and shown in Table 6.

**Table 6:** Political empowerment of farmers through e-Agriculture

Sl. no.	Political empowerment components	Mean score		t test value
		Before e-Agriculture use	Before e-Agriculture use	
1.	Participation in social well-being activities	2.15	3.10	14.661**
2.	Membership in the social organization	2.24	3.24	5.813**
3.	Freedom of expression	1.80	2.99	5.415**
4.	Conflict management	1.90	3.93	6.574**
Total mean score		9.96	16.25	
Overall mean score		1.99	3.25	

** Significance at the level of 1 % (t-value)

Table 6 revealed that the mean scores obtained by the respondents in political empowerment components, before and after the usages of e-Agriculture had also improved. With respect to conflict management, the average score obtained by the respondents was increased the highest by 1.22. Rest of other variables, such participation in social well-being activities, membership in social organization, an improvement had been recorded. Data showed that, the t test also supported the obtained mean score and shown a significant difference.

Since the political empowerment factor was positively significant to empower the farmers, the researchers investigated further to identify the knowledge empowerment factor.

### Knowledge empowerment

The knowledge empowerment was analyzed in terms of awareness of information, knowledge and skills possessed by the respondents before and after usages of e-Agriculture presented in Table 7.

**Table 7:** Knowledge empowerment of farmers through e-Agriculture

Sl. no.	Knowledge empowerment components	Mean score		t test value
		Before e-Agriculture use	After e-Agriculture use	
1.	Use of machineries and equipments	2.32	3.26	12.109**
2.	Knowledge on value addition	1.47	2.67	2.813**
3.	Adoption of IPM practices	1.99	3.29	5.614**
4.	Adoption of INM practices	1.01	2.20	1.620
5.	Adoption of IWM practices	1.12	2.35	0.896
Total mean score		7.91	13.77	
Overall mean score		1.582	2.754	

** Significance at the level of 1 % (t-value)

It was noticed (Table 7) that adoption of integrated farm management practices (IPM, INM and IWM) by the respondents had been increased from 1.99, 1.01, and 1.12 to 3.29, 2.20, and 2.35 respectively after usages of e-Agriculture. All the respondents (100 %) had responded positively when asked questions regarding knowledge on the use of machinery and equipment after usages of e-Agriculture. Adoption of IPM practices (CMD 1.31) has been contributed heavily to the knowledge empowerment dimension. It was evident that mean scores of all the dimensions of empowerment were increased greatly after usages of e-Agriculture. The major reason for knowledge empowerment was mainly due to their participation in the digital video programs conducted by various specialists to the respected field. The t test value also supported the obtained mean score and shown a significant difference.

Since the knowledge empowerment factor was positively significant to empower the farmers, the researchers investigated further to identify the psychological empowerment factor.

### Psychological empowerment

The psychological empowerment of the farmers was assessed in terms of change in motivation in farming, decision making quality, risk taking ability etc. and furnished in Table 8.

**Table 8:** Psychological empowerment of farmers through e-Agriculture

Sl. no.	Psychological empowerment components	Mean scores		t test value
		Before e-Agriculture use	After e-Agriculture use	
1.	Motivation in farming	2.15	3.10	12.894**
2.	Self esteem	2.24	3.24	10.864**
3.	Risk taking ability	1.80	2.99	5.575**
4.	Confidence	1.90	3.93	6.574**
5.	Decision making ability	1.87	2.99	5.816**
Total mean score		9.96	16.25	
Overall mean score		1.99	3.25	

** Significance at the level of 1 % (t-value)

Table 8 revealed that there had been considerable improvement in the psychological attributes of the respondents. Remarkable improvement in confidence was noticed (mean score from 1.9 to 3.93). The risk taking ability of the members had also been increased. With regard to feeling of positive attitude and self-esteem and decision making ability, there had been an outstanding improvement, were noticed. Self-esteem had been increased from the mean score of 2.24–3.24 respectively. The t test also supported the obtained mean score and shown significant difference.

From the above discussion the studies revealed that economic empowerment, family and social empowerment, political empowerment, knowledge empowerment and psychological empowerment were positively impact on farmers empowerment, so empowerment was calculated using the following formula:

$${\text{EoF}} = {\text{Eeco}} + {\text{Efs}} + {\text{Epol}} + {\text{Ekno}} + {\text{Epsy}}$$

where, EoF = Empowerment of farmers, Eeco = Economic empowerment, Efs = Family and social empowerment, Epol = Political empowerment, Ekno = Knowledge empowerment and Epsy = Psychological empowerment.

From the final empowerment were also studied:

(1) Is e-Agriculture substantially contributed towards farmers’ empowerment? And (2) among the factors which factor was significantly changed the empowerment of the study group due to the involvement of e-Agriculture?

In the next segment of the study, analysis was carried forward to identify the highest significantly contributing factors regarding farmers’ empowerment.

## Variables contributing in farmers’ empowerment

For this study twelve characteristics of the respondent were selected and each of the characteristics was treated as independent variable. The selected characteristics were age (X_1_), education (X_2_), farm size (X_3_), annual household income(X_4_), farming experience (X_5_), participation in training (X_6_), agricultural knowledge (X_7_), usages of e-Agriculture (X_8_), attitude towards e-Agriculture (X_9_), organizational participation (X_10_), cosmopoliteness (X_11_) and availability of e-Agriculture (X_12_). Empowerment through e-Agriculture (Y) was the dependent variable of this study. The final null hypothesis: There is no contribution of the selected characteristics (age, education, farm size, annual household income, farming experience, participation in training, agricultural knowledge, usages of e-Agriculture, attitude towards e-Agriculture, organizational participation, cosmopoliteness, availability of e-Agriculture) of farmers in empowering them through e-Agriculture.

In order to avoid the misleading results and to determine the best explanatory variables, the method of stepwise multiple regressions was administrated and 8 independent variables were fitted together in step-wise multiple regression analysis. Table 9 shows the summarized results of step-wise multiple regression analysis with 8 independent variables on empowerment through e-Agriculture. It was observed that out of 8 variables only 5 independent variables namely farm size (X_3_), usages of e-Agriculture (X_8_), attitude towards e-Agriculture (X_9_), organizational participation (X_10_), and cosmopoliteness (X_11_) were entered into the regression equation which contribute the farmers empowerment. The other three variables were not entered into regression equation. The regression equation so obtained is presented below:

$${\text{Y}} = 10.75 + 0.066{\text{X}}_{3} + 0.450{\text{X}}_{8} + 0.250{\text{X}}_{9} + 0.137{\text{X}}_{10} + 0.180{\text{X}}_{11}$$

The multiple R and R^2^ values were found 0.796 and 0.892 respectively and the corresponding F-ratio was 201.782 which were significant at 0.000 levels. For determining unique contribution of each of the five variables the increase in R^2^ value was determined on empowerment. These five variables jointly explained 88.4 % of the total variation in empowering farmers through e-Agriculture. Usages of e-Agriculture alone contributed 83.4 % of the variation followed by attitude towards e-Agriculture (3.3 %), organizational participation (0.9 %), cosmopoliteness (0.4 %) and farm size (0.04 %) variation in empowering farmers through e-Agriculture. In summary, the models suggest that the respective authority should consider the respondents’ usages of e-Agriculture, attitude towards e-Agriculture, organizational participation, cosmopoliteness and farm size.

**Table 9:** Summary of step wise multiple regression analysis showing the contribution of selected characteristics of the respondents to empower them through e-Agriculture

Variables entered	Standardized partial ‘b’ coefficients	Value of ‘t’ (with probability level)	Adjusted R^2^	Increase in R^2^	Variation explained in percent
Usages of e-Agriculture (X_8_)	0.450	6.319 (000)	0.834	0.834	83.4
Attitude towards e-Agriculture (X_9_)	0.250	4.280 (000)	0.867	0.033	3.3
Organizational participation (X_10_)	0.137	2.856 (005)	0.876	0.009	0.9
Cosmopoliteness (X_11_)	0.180	2.615 (0.010)	0.880	0.004	0.4
Farm size (X_3_)	0.066	2.172 (0.032)	0.884	0.004	0.4
Total				0.884	88.4

R-square = 0.892

Adjusted R-square = 0.885

F-ratio = 201.782

Standard error of estimate = 2.01

Constant = 10.75

## Conclusion

This study suggested that e-agriculture had positively significant impact on the farmers’ empowerment of Bangladesh. In addition to that, the study also revealed that the factors i.e. usages of e-Agriculture, attitude towards e-Agriculture, organizational participation, cosmopoliteness and farm size were contributed to change farmers empowerment significantly due to the involvement of e-Agriculture. Finally, it indicated that usage of e-Agriculture alone contributed 84 % of the variation of empowerment. Based on these findings, the researchers would like to suggest two policy level implications. 1) The government should implement such e-auricular projects on a larger scale all over the country 2) To popularize this service, government should implement integrated marketing communication using the popular print and electronic media so that more and more people get aware of this service.
